# Rare Cardiovascular Disease Care: Centers of Excellence or Excellence of Centers?

**DOI:** 10.1016/j.jaccas.2023.101891

**Published:** 2023-08-02

**Authors:** Nowell M. Fine, Karan Shahi

**Affiliations:** Division of Cardiology, Department of Cardiac Sciences, Libin Cardiovascular Institute, Cumming School of Medicine, University of Calgary, Calgary Alberta, Canada

**Figure:**
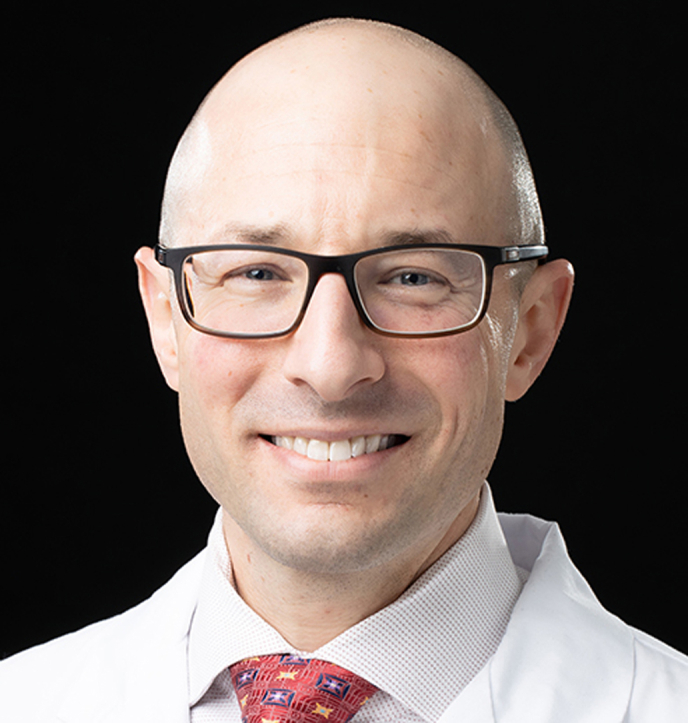

The term “center of excellence” in health care is often poorly defined and frequently overused.^1^^,^^2^ In some medical fields, this term has clearly established and detailed criteria relating to accreditation or certification by a medical society or other organization or agency for a specific condition or disease and/or invasive procedure.^3^ In others, the term is used more loosely without any supporting criteria or standards, either by a single center or group of centers, and sometimes for the purpose of self-promotion to attract patients. Despite this, the term has been used for many years in health care and is likely here to stay. The value of designating centers of excellence for both patients and referring clinicians has at times been controversial and debated. Although few would argue that establishing quality-of-care standards and benchmarks serves to benefit patients, some may feel that such labels do not acknowledge the care provided in centers that are not recognized as “excellent” and may unfairly lead to the perception of tiered health care. However, it is also recognized that even for common conditions and procedures, centers with high patient care volumes and dedicated expertise often play an important role in advancing the field and contributing to the establishment of quality-of-care standards, among other benefits.^1^, ^2^, ^3^ Arguably, perhaps greater utility for having established centers of excellence is for rare conditions and/or less commonly performed (or particularly technically difficult) procedures. Their value in these settings is not hard to understand, because rare diseases naturally have a lower level of awareness among clinicians, which consequently may result in significant delays in diagnosis that may be at the expense of disease progression and organ damage.^4^ However, establishing consensus criteria for defining a center of excellence and implementing such standards into practice in medical centers at large may present more practical challenges.

Beyond the more obvious advantages of dedicated experience for diseases that often have very specific and nuanced elements of management, there are other factors that may increase the value and importance of defining centers of excellence for rare cardiovascular disease care. For example, often such conditions require specialized (and frequently costly) diagnostic testing approaches that simply may not be available in other locations. Similarly, many rare cardiovascular diseases have multisystem manifestations and therefore require care from multiple medical disciplines and specialties that may also be unavailable in many centers.^1^^,^^4^ These infrastructure elements can be challenging to acquire, even for larger medical centers, and often take years to build and develop through the efforts of dedicated clinicians.

When considering the delivery of care for rare cardiovascular diseases, 2 important principles that may be at odds with each other are the value of standardized approaches and criteria for defining them versus the unique settings in which optimal care delivery may be achieved, even among larger medical centers. Put another way, one size may not fit all, and organizations that endeavor to formally define centers of excellence for rare cardiovascular diseases need to cognizant of this to ensure that criteria are not overly restrictive or tailored to suit certain centers. Geography is an example of a factor that is often important regarding care delivery for rare conditions, and in particular in urban vs rural areas.^3^ Large urban medical centers often have a resource and infrastructure advantage for developing a rare disease center of excellence, and for this reason, multiple specialized centers may arise in close proximity of each other and even compete for a small population of patients from within their own region. Whereas competition among centers is not necessarily bad and may foster advancements, patients who live remotely to these centers are challenged by traveling (sometimes long distances) to receive the care they need. Such clustering of expertise may be caused by several factors, one of which being that dedicated training in rare conditions is only offered in select centers and may influence where trainees ultimately decide to practice. Furthermore, even larger centers may lack personnel with the specialized expertise needed to provide optimal care for some rare diseases. Attempting to evenly distribute centers of excellence geographically would be challenging for several reasons; however, when possible, ideally this would be considered by medical societies or other organizations when evaluating center of excellence designations. Such considerations may also be dictated by disease characteristics and regional prevalence, especially for hereditary diseases that may have endemic areas and/or founder effect regions. Other access to care challenges for patients may include health care coverage, especially for medications that may be very costly for some rare cardiovascular diseases (amyloidosis and Anderson-Fabry disease being 2 frequently referenced examples), and treating centers frequently have experience needed to advocate and assist patients who lack the coverage required.^4^

In addition to some of the structural criteria for defining a center of excellence just discussed, such as patient volume and infrastructure, what other characteristics and metrics might be important to consider? A number of publications have attempted to provide some general criteria for defining a center of excellence that could be applied broadly across different diseases. These include, but are not limited to, collecting and reported measures of patient outcomes (typically applied more for surgical or procedural therapies); participation in clinical trials and patient registries or other longitudinal studies that may be either quality focused or research oriented (and in some cases such registries also provide outcomes that can be compared and benchmarked to establish standards); a clearly defined governance structure and organization of leadership and administrative supports, which often includes a commitment to shared decision making among the multiple disciplines and specialties involved in the care of rare cardiovascular diseases, inclusive of patients and families/caregivers; processes in place for performance of formal quality assessment and improvement initiative(s); and participation in various research, education, and advocacy activities and initiatives.^1^^,^^3^^,^^4^

The educational role of centers of excellence in rare cardiovascular diseases is of particular importance. Beyond simply providing “excellent” rare disease care, these centers also have a responsibility to educate and train health care professionals outside of their institution on the management of the condition, especially for those within their referral region.^4^ This is to ensure that patients who must travel to receive specialized care for a rare disease also have access to care in their area if needed. Such an approach has been referred to as the “hub-and-spoke” model that is often used in fields such as transplant medicine, whereby local clinics are supported by specialized centers. Depending on the disease and region, education of such regional centers may ideally progress to the level of independence (where possible) in providing rare disease care to improve access for patients. Whereas improvements and greater acceptance of “virtual medicine” strategies has made it easier for patients to receive care from a distance, there will always be a critical role for communication and building relationships between referral and local centers. Education of patients, their families, and the public about the disease is also an important responsibility of such centers. This also involves close collaboration with organizations that are dedicated to rare diseases such as patient support groups and other advocacy groups.

Another challenge in prescribing best practice recommendations for rare disease care is the unique nature of each condition. Although this paper refers to “rare cardiovascular diseases” in general terms, in fact these conditions can be very different with distinctive manifestations, incidence and prevalence, diagnostic approaches, management strategies, and prognosis. Acknowledging this, having criteria and standards in place to establish centers of excellence has a role for advancing patient care practices and remains an important priority for rare disease organizations and societies. Commensurate with this is the role of rare disease referral centers in educating those outside of their center and facilitating conditions that improve access and advocacy for all patients. Through this work, those providing rare cardiovascular disease care can work together to build networks that offer patients both centers of excellence and excellence within referring centers.

## Funding Support and Author Disclosures

The authors have reported that they have no relationships relevant to the contents of this paper to disclose.
